# Human Superantibodies to 3CL^pro^ Inhibit Replication of SARS-CoV-2 across Variants

**DOI:** 10.3390/ijms23126587

**Published:** 2022-06-13

**Authors:** Kittirat Glab-ampai, Kanasap Kaewchim, Thanatsaran Saenlom, Watayagorn Thepsawat, Kodchakorn Mahasongkram, Nitat Sookrung, Wanpen Chaicumpa, Monrat Chulanetra

**Affiliations:** 1 Center of Research Excellence in Therapeutic Proteins and Antibody Engineering, Department of Parasitology, Faculty of Medicine Siriraj Hospital, Mahidol University, Bangkok 10700, Thailand; kittirat.gla@mahidol.edu (K.G.-a.); kanasap.kaw@alumni.mahidol.ac.th (K.K.); thanatsaran.sae@mahidol.edu (T.S.); watayagorn.the@mahidol.edu (W.T.); kodchakorn.mah@mahidol.ac.th (K.M.); nitat.soo@mahidol.ac.th (N.S.); wanpen.cha@mahidol.ac.th (W.C.); 2 Graduate Program in Immunology, Department of Immunology, Faculty of Medicine Siriraj Hospital, Mahidol University, Bangkok 10700, Thailand; 3 Biomedical Research Incubator Unit, Department of Research, Faculty of Medicine Siriraj Hospital, Mahidol University, Bangkok 10700, Thailand

**Keywords:** SARS-CoV-2, major protease (3CL^pro^), human single-chain antibody variable fragments (HuscFvs), cell-penetrating antibody, superantibody

## Abstract

Broadly effective and safe anti-coronavirus agent is existentially needed. Major protease (3CL^pro^) is a highly conserved enzyme of betacoronaviruses. The enzyme plays pivotal role in the virus replication cycle. Thus, it is a good target of a broadly effective anti-*Betacoronavirus* agent. In this study, human single-chain antibodies (HuscFvs) of the SARS-CoV-2 3CL^pro^ were generated using phage display technology. The 3CL^pro^-bound phages were used to infect *Escherichia coli* host for the production the 3CL^pro^-bound HuscFvs. Computerized simulation was used to guide the selection of the phage infected-*E. coli* clones that produced HuscFvs with the 3CL^pro^ inhibitory potential. HuscFvs of three phage infected-*E. coli* clones were predicted to form contact interface with residues for 3CL^pro^ catalytic activity, substrate binding, and homodimerization. These HuscFvs were linked to a cell-penetrating peptide to make them cell-penetrable, i.e., became superantibodies. The superantibodies blocked the 3CL^pro^ activity in vitro, were not toxic to human cells, traversed across membrane of 3CL^pro^-expressing cells to co-localize with the intracellular 3CL^pro^ and most of all, they inhibited replication of authentic SARS-CoV-2 Wuhan wild type and α, β, δ, and Omicron variants that were tested. The superantibodies should be investigated further towards clinical application as a safe and broadly effective anti-*Betacoronavirus* agent.

## 1. Introduction

Severe acute respiratory syndrome coronavirus 2 (SARS-CoV-2) is the causative agent of the on-going coronavirus disease 19 (COVID-19) pandemic that initially broke out in Wuhan, China, in December 2019 [1]. The SARS-CoV-2 is an enveloped, positive sense, single stranded RNA virus that taxonomically belongs to the order Nidovirales, family Coronaviridae, subfamily Coronavirinae, and genus *Betacoronavirus* [2]. The SARS-CoV-2 virion uses a receptor binding domain (RBD) located in the S1 subunit of the surface-exposed trimeric spike (S) glycoprotein to bind to the human angiotensin-converting enzyme 2 (hACE2) receptor (the same receptor as for SARS-CoV) for host cell entering and replicating therein [3]. This process requires host membrane proteases to cleave the S protein at the junction of S1-S2 subunits and S2′ site [4]. After host-viral membrane fusion mediated by the conformationally rearranging S2 subunit components [fusion peptide (FP), heptad repeat (HR) 1 and HR2], the virus RNA genome is released into the cytosol [5]. Other molecules on the hACE2 expressing host cells including heparan sulfate, sialic acids, neuropillin-1 (NRP1), CD147 and glucose-regulated protein 78 (GRP78) may participate in the virus entry [6]. Within the cytosol, the open reading frames ORF1a and ORF1b located at the 5′-two-thirds of the viral genome translate into two polyproteins, pp1a and pp1ab, which are then cleaved by the virus proteases into 16 mature non-structural proteins with different functions [7]. The virus uses a rough endoplasmic reticulum membrane to form RNA replicase-transcriptase complex for synthesizing minus-sense RNAs, which transcribe to full-length genomic, as well as canonical subgenomic (sg) RNAs that code for the viral structural and accessory proteins. The genes coding for the virus structural and accessory proteins are located at the 3′-one-third of the genome. The newly synthesized full-length viral RNA and the translated structural proteins and some accessory proteins (p3a, p7a, p7b, p9b) are assembled into progeny viruses in the ER–Golgi intermediate compartment (ERGIC) and are released by exocytosis [8].

Chymotrypsin-like cysteine protease (3CL^pro^) plays an important role in the *cis*- and *trans*-cleavage of the pp1a and pp1ab polyproteins at 11 sites during the early stage of the coronavirus replication cycle; thus, the enzyme is also called major protease (M^pro^) [9]. The 3CL^pro^ protein acquires active protease activity only after forming homodimer [10,11]. Molecularly, the 3CL^pro^ monomer is composed of three domains: I, II and III (residues 8–101, 102–184, and 201–303, respectively) [12,13]. The catalytic site of the 3CL^pro^ formed by His41 and Cys145 is located in the cleft between domains I and II [14,15]. The Cys145 thiol functions as the nucleophile in the proteolytic process [13,16]. The 3CL^pro^ catalytic active site contains S1′, S1, S2, and S4 [13]. The 3CL^pro^ substrate has a glutamine (Glu/Q) in position P1 (the position just before the cleavage site) and serine (Ser/S) in position P1′ (the position just after the cleavage site) [17,18]. There is a cluster of conserved serine residues (Ser139, Ser144, and Ser147) in a proximity of the 3CL^pro^ active site; Ala substitutions of these Ser residues dampened the 3CL^pro^ catalytic activity [19]. Besides, mutation of Ser147 leads to 3CL^pro^ dimer instability, which renders the enzyme inactive [20]. The 3CL^pro^ domain I and domain II contain a substrate-binding site [16,21]. A long loop region (residues 185–200) connects domains II and III [22]. The domain III (residues 201−303) regulates dimerization of 3CL^pro^ into active protease [12]. Residues involved in the 3CL^pro^ dimerization are Arg4, Ser10, Gly11, Glu14, Asn28, Ser139, Phe140, Ser147, Glu290, and Arg298 [12]. Amino-terminal residues 1–7 (N-finger) of one monomer in the 3CL^pro^ dyad interact with Glu166 of the other monomer and this keeps the S1 pocket of the substrate-binding site in the correct orientation [12].

The 3CL^pro^ is highly conserved among the members of genus *Betacoronavirus* including SARS-CoV, MERS-CoV, Bat CoVs and SARS-CoV-2 and plays a pivotal role in the early stage of the coronavirus replication cycle. Besides, there is no human homolog of this protein [12]. Therefore, the 3CL^pro^ is an attractive target of broadly effective anti-coronavirus agents. A variety of small molecular pharmacological inhibitors and plant derived drugs have been investigated for anti-SARS-CoV-2 treatment [9,23,24,25,26,27,28,29,30]. In this study, we generated cell-penetrable fully human single-chain antibodies (human superantibodies) that bound to intracellular 3CL^pro^. The superantibodies inhibited replication of the SARS-CoV-2 across Wuhan wild type and the mutated descendants. They should be developed further towards clinical application as a mutation-resistant, broadly effective, and safe therapeutic agent against the SARS-CoV-2, and possibly also against other coronaviruses.

## 2. Result

### 2.1. Production of Recombinant 3CL^pro^ (r3CL^pro^) of SARS-CoV-2

**Figure 1:**
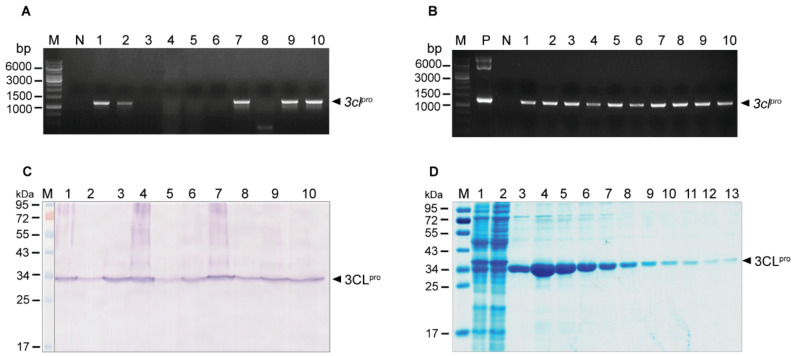
Preparation of recombinant 3CL^pro^ of SARS-CoV-2. (**A**) Amplicons of *3cl^pro^* amplified from *3cl^pro^*-pTriEx1.1-transformed DH5α *E. coli* clones. Lane M 1 kb DNA ladder; lane N, negative control (no DNA template); lanes 1, 2, 7, 9 and 10 show *3cl^pro^* amplicons at ~1300 base pairs (bp) (arrowhead) that were amplified from DH5α *E. coli* clones 1, 2, 7, 9 and 10, respectively. Transformed DH5α *E. coli* clones 3–6 and 8 were negative for the recombinant vector. (**B**) Amplicons of *3cl^pro^* amplified by using colonies of NiCo21 (DE3) *E. coli* as the direct DNA templates. Lane M, 1 kb DNA ladder; lane N, negative control (no DNA template); lane P, positive control; lanes 1–10, transformed NiCo21 (DE3) *E. coli* clones 1–10, respectively. Numbers at the left of (**A**,**B**) are DNA sizes in base pairs. (**C**) Recombinant 3CL^pro^ produced by transformed NiCo21 (DE3) *E. coli* clones that contained recombinant *3cl^pro^*-pTriEx1.1 vector. Lane M, pre-stained protein ladder; lanes 1–10, rCL^pro^ (~34 kD, arrowhead) of 10 NiCo21 (DE3) *E. coli* clones. (**D**) Purified recombinant 3CL^pro^ after SDS-PAGE and CBB staining. Lane M, pre-stained protein ladder; lane 1, crude lysate of transformed NiCo21 (DE3) *E. coli*; lane 2, flow through fraction; lanes 3–12, 150 mM imidazole eluted fractions 1–10, respectively; lane 13, 1 M imidazole eluted fraction. Numbers at the left of (**C**,**D**) are protein masses in kDa.

The recombinant 3CL^pro^ of SARS-CoV-2 with active inherent protease activity was produced and used as an antigen in the phage panning to select out the 3CL^pro^-bound phages from the HuscFv phage display library. For production of the SARS-CoV-2 r3CL^pro^, the 3CL^pro^ gene (*3cl^pro^*) was subcloned from the commercially synthesized *3cl^pro^*-pET23a+ to pTriEx1.1 expression plasmid and the recombinant *3cl^pro^-*pTriEx1.1 DNA was transformed into DH5α *Escherichia coli*. Figure 1A shows *3cl^pro^* amplicons from several transformed DH5α *E. coli*. The recombinant plasmids from one of the transformed DH5α *E. coli* colonies were subsequently introduced to NiCo21 (DE3) *E. coli.* Figure 1B shows amplicons of the *3cl^pro^* amplified from different *3cl^pro^*-pTriEx1.1 transformed NiCo21 (DE3) *E. coli* clones. These transformed *E. coli* clones readily expressed r3CL^pro^ (~34 kDa), as shown in Figure 1C. The 6× His tagged-r3CL^pro^ was purified from homogenate of one of the transformed NiCo21 (DE3) *E. coli* clones by using TALON^®^ Metal Affinity resin (Thermo Fisher Scientific, Waltham, MA, USA); the resin-bound recombinant 3CL^pro^ was eluted with 150 mM imidazole solution into 1-mL fractions and subjected to SDS-PAGE and Coomassie Brilliant Blue G-250 (CBB) staining (Figure 1D).

### 2.2. Protease Activity of Recombinant 3CL^pro^ Determined by Fluorescence Resonance Energy Transfer (FRET) Assay

The Fluorescence Resonance Energy Transfer (FRET) assay was based on a 58-kDa 3CL^pro^ substrate that contained two fluorescent proteins, i.e., cyan fluorescent protein (CFP) and yellow fluorescent protein (YFP) interconnected via an autocleavage sequence of 3CL^pro^ (TSAVLQ↓SGFRKM which are the P6P5P4P3P2P1↓P1′P2′P3′P4′P5P6′ sites, respectively) [31]. When the CFP in the substrate is excited at ~430 nm, the YFP emits fluorescence at ~530 nm through FRET. Cleavage of the peptide bond at TSAVLQ↓SGFRKM by active 3CL^pro^ separates the CFP and YFP, leading to a decrease in the emitted fluorescence at ~530 nm after the CFP excitation. After digestion by the active 3CL^pro^, the 58-kDa substrate produced 28 and 30 kDa products [31]. In the presence of the 3CL^pro^ inhibitor, the substrate cannot be cleaved; thus, high fluorescence emission was detected after the CFP was excited. 

**Figure 2:**
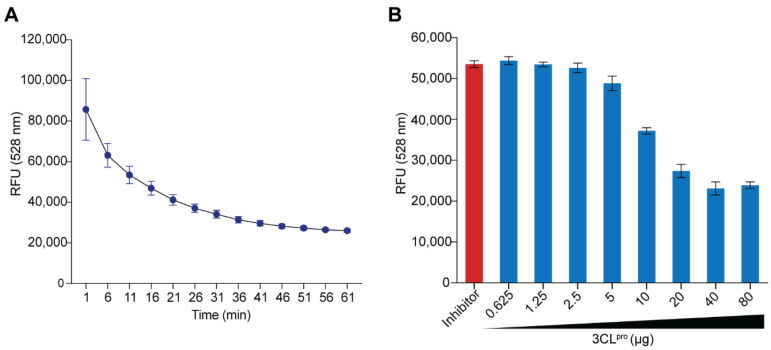
Enzymatic activity of the r3CL^pro^ tested by FRET assay using *E. coli*-derived recombinant CFP-TSAVLQ↓SGFRKM-YFP fusion protein as the fluorogenic substrate. (**A**) The r3CL^pro^ (80 μg) was mixed with 5 μg of the fluorogenic substrate and kept at 25 °C for 60 min; the emitted relative fluorescence units (RFU) at 528 nm were measured at 5 min-intervals with the CFP excitation wavelength. The active 3CL^pro^ cleaved the fluorogenic substrate resulted in a decrease of the emitted RFU at 528 nm (*y*-axis) in a time-dependent manner (*x*-axis). (**B**) Various amounts of r3CL^pro^ (0.625–80 μg) were mixed with 5 μg of the fluorogenic substrate and incubated at 25 °C for 4 h. The r3CL^pro^ cleaved the substrate in a dose-dependent manner. Inhibitor, r3CL^Pro^ mixed with protease inhibitor of Untagged SARS-CoV-2 3CL Protease assay kit (BPS Bioscience) before adding the substrate.

The purified *E. coli*-derived r3CL^pro^ showed enzymatic activity when tested with the purified recombinant fluorogenic substrate in the FRET assay, as shown in Figure 2. In this experiment, 80 μg of the r3CL^pro^ was mixed with 5 μg of the fluorogenic substrate and the reaction mixture was kept at 25 °C for 60 min. The emitted relative fluorescence units (RFU) at 528 nm were measured at 10 min-intervals. The r3CL^pro^ cleaved the substrate in a time-dependent manner (Figure 2A). When various amounts of the r3CL^pro^ (0.625–80 μg) were mixed with a fixed amount of the substrate (5 μg) and kept at 25 °C for 4 h, substrate cleavage was enzyme dose-dependent (Figure 2B).

### 2.3. Human Single-Chain Antibodies (HuscFvs) to r3CL^pro^ Prepared by Phage Display Technology

**Figure 3:**
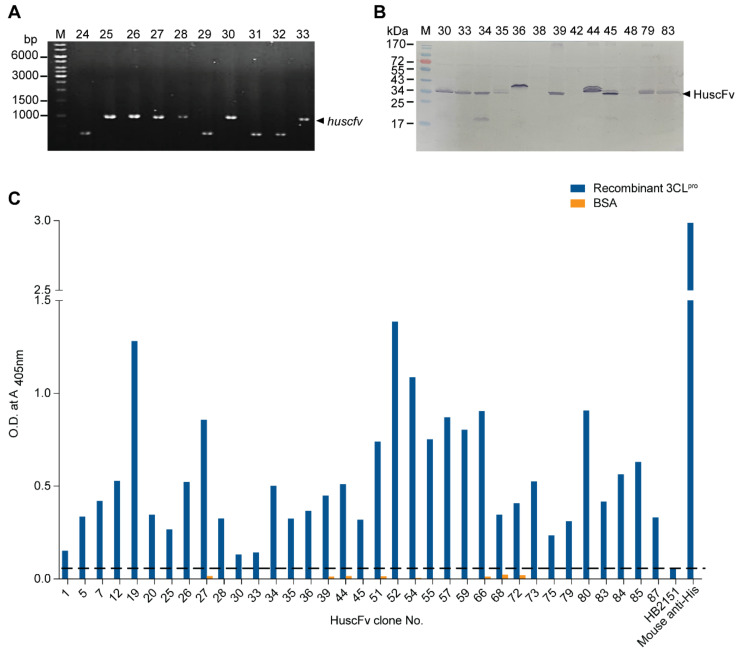
HuscFvs that bound to r3CL^pro^ by indirect ELISA. (**A**) Amplicons of HuscFv genes (*huscfvs*;) amplified from representative phage transformed-HB2151 *E. coli* colonies by direct colony PCR. Lane M, standard DNA marker; lanes 25–28, 30, and 33 show *huscfv* amplicons (1000 bp, arrowhead). Numbers at the left are DNA sizes in bp. (**B**) Western blot patterns of E-tagged-HuscFvs in lysates of representative *huscfv*-positive HB2151 *E. coli* clones (the expressed HuscFvs from some *E. coli* clones appeared as protein doublets at about 34–36 kDa; upper band, immature HuscFv with signal peptide; lower band, mature HuscFvs without signal peptide). (**C**) HuscFvs in lysates of 35 HB2151 *E. coli* clones (clones 1, 5, 7, 12, 19, 20, 25, 26, 27, 28, 30, 33, 34, 35, 36, 39, 44, 45, 51, 52, 54, 55, 57, 59, 66, 68, 72, 73, 75, 79, 80, 83, 84, 85 and 87) bound to r3CL^pro^ and gave significant ELISA signals [optical density (OD) 405 nm to r3CL^pro^ at least two times higher than the same lysate to the control antigen, BSA]. Broken line, arbitrarily cut-off OD 405 nm of the indirect ELISA. HB2151, lysate of original HB2151 *E. coli* used as negative (no antibody) binding control. Mouse anti-His was used as positive binding control (bound to 6× His 3CL^pro^).

The enzymatically active r3CL^pro^ was used as antigenic bait in the phage panning process for fishing out the r3CL^pro^-bound phages from the HuscFv phage display library [32]. The HB2151 *E. coli* infected with the r3CL^pro^-bound phages that grew on the selected agar plates (91 colonies) were screened for the clones that carried the HuscFv genes (*huscfvs;* 1000 bp) by direct colony PCR using the phagemid specific primers (R1 and R2); 53 clones (~58%) were positive. Figure 3A shows *huscfv* amplicons amplified by direct colony PCR from representative phage transformed HB2151 *E. coli* clones. These *huscfv*-positive clones produced HuscFvs (~34–36 kDa) which the representatives are shown in Figure 3B. Among the 53 clones, HuscFvs in lysates of 35 clones bound to the purified 6× His tagged-r3CL^pro^ by indirect ELISA (Figure 3C).

### 2.4. Computerized Simulation to Guide Selection of the HuscFv-Producing E. coli Clones

**Figure 4:**
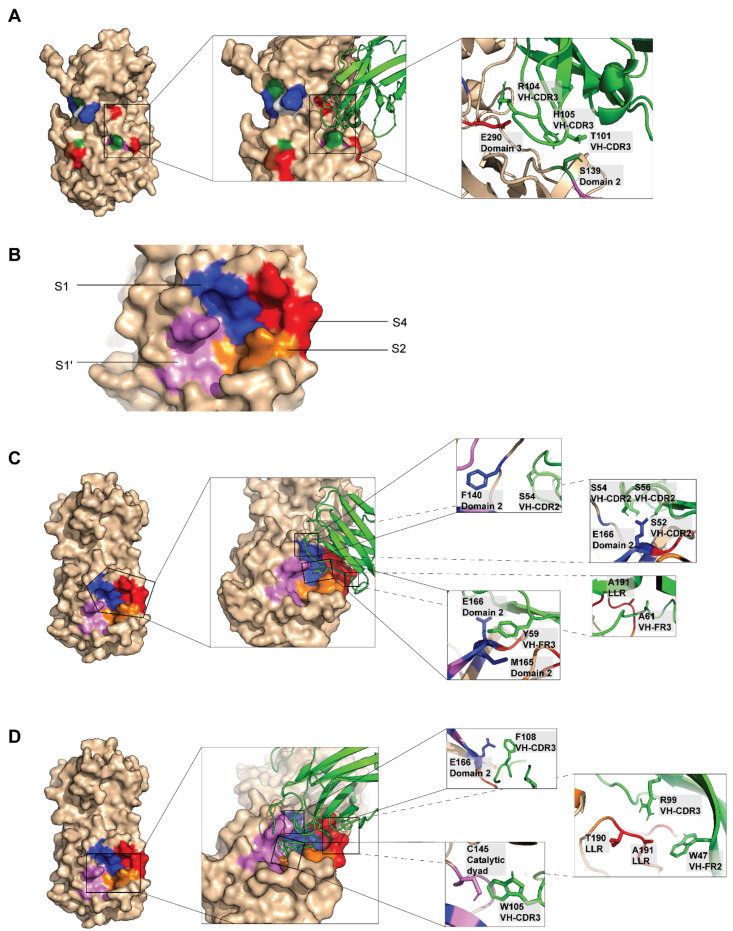
Computerized homology modeling and intermolecular docking of HuscFvs and 3CL^pro^. (**A**) Left panel is three-dimensional structure of monomeric 3CL^pro^ (ivory) showing important residues for homodimerization. The amino acids are shown in CINEMA color scheme: polar negative Asp and Glu are red; polar positive His, Lys, and Arg are blue; polar neutral Ser, Thr, Asn and Gln are green; non-polar aromatic Phe and Tyr are magenta; non-polar aliphatic Ala, Val, Leu, Ile, and Met are white; Pro and Gly are brown); middle panel of (**A**) shows contact interface between 3CL^pro^ and HuscFv27 (green); right panel of (**A**) shows important interacting residues of 3CL^pro^ and HuscFv27. (**B**) Highlight the substrate-binding sites, i.e., S1 (blue), S1′ (violet), S2 (orange) and S4 (red) of the 3CL^pro^ (ivory). The S3 (E166) cannot be seen as it is located under the S1. (**C**) Left panel shows surface of 3CL^pro^ (blue, red, orange and pink) that formed contact interface with HuscFv33; middle panel shows contact interface between 3CL^pro^ and HuscFv33 (green); right panel shows important interacting residues of 3CL^pro^ and HuscFv33. (**D**) Left panel shows surface of 3CL^pro^ (blue, red, orange and pink) that forms contact interface with HuscFv34; middle panel shows contact interface between 3CL^pro^ and HuscFv34 (green); right panel shows important interacting residues of 3CL^pro^ and HuscFv34.

Nucleotides sequences of the *huscfvs* of the 35 HB2151 *E. coli* clones that expressed r3CL^pro^ bound-HuscFvs were sequenced and deduced into amino acid sequences by using the IMGT/V-QUEST analytical tool. Amino acid sequences of 22 *E. coli* clones (clones 5, 12, 19, 25, 26, 27, 33, 34, 35, 36, 39, 44, 45, 51, 52, 55, 72, 73, 75, 80, 83 and 84) were complete single-chain antibody sequences, i.e., each of their VH and VL domains contained three CDRs (CDR1-3) and four FRs (FR1-4) with the (Gly_4_Ser)_3_ linker between the two domains, based on the International Immunogenetics Information Systems. Thus, the 3D structures of these 22 clones were docked with the 3CL^pro^ 3D structure to guide selection of the HuscFvs with 3CL^pro^ inhibitory potential. Among HuscFvs of the 22 *E. coli* clones, HuscFvs of three clones, 27, 33 and 34, bound presumptively to residues/regions of the 3CL^pro^ that are critical for the enzymatic activity. In-silico docking predicted that the HuscFv27 interacted with Ser139 of the catalytic site (Figure 4A). This residue functions also in 3CL^pro^ dimerization to form a functionally active enzyme. The HuscFv27 also interacted with another dimerization residue, Glu290 of the 3CL^pro^ (Figure 4A). Figure 4B shows substrate-binding sites (S1′, S1, S2, and S4) of the 3CL^pro^. The S3 site cannot be seen as it is under the S1. HuscFv33 and HuscFv34 interacted presumptively with the substrate-binding pocket of the 3CL^pro^ [33]. The HuscFv33 interacted with Met165 and Ala191 (Figure 4C), while the HuscFv34 interacted with Cys145 of the enzyme catalytic dyad, as well as Glu166, Thr190 and Ala191 (Figure 4D). Moreover, the HuscFv33 and HuscFv34 also interacted with the 3CL^pro^ dimerization residues, i.e., Glu166 and Phe140 for HuscFv33 (Figure 4C) and Glu166 for HuscFv34 (Figure 4D). Table 1 gives details of the predicted residues, residue functions and domains of 3CL^pro^, and chemical bonds involved in the 3CL^pro^-HuscFvs interactions.

### 2.5. Cell-Penetrating Peptide (CPP)-Linked HuscFvs

**Figure 5:**
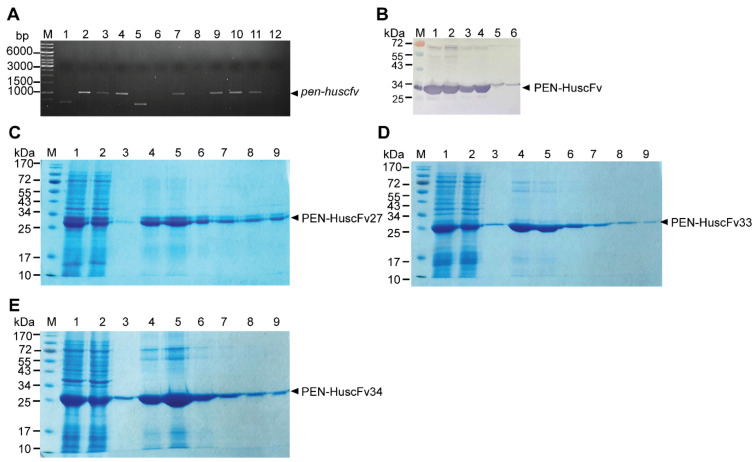
Production of cell-penetrable HuscFvs (penetratin-linked HuscFvs/PEN-HuscFvs). (**A**) Amplicons of *pen-huscfvs* (arrowhead) from DH5α *E. coli* clones that were transformed with *pen-huscfv*-pET23b+ vector. Lane M, 1 kb DNA ladder. Lanes 1–3, *pen-huscfv27*-pET23b+ transformed DH5α *E. coli* clones 1–3, respectively; clones 2 and 3 (lanes 2 and 3) were positive for *pen-huscfv27* amplicons. Lanes 4–8, *pen-huscfv33-*pET23b+ transformed DH5α *E. coli* clones 1–4, respectively; clones 1 and 4 (lanes 4 and 7) were positive for *pen-huscfv33* amplicons. Lanes 9–11, *pen-huscfv34*-pET23b+ transformed DH5α *E. coli* clones 1–5, respectively; clones 2–4 (lanes 9–11) were positive for *pen-huscfv34*. Numbers at the left are DNA sizes in bp. (**B**) PEN-HuscFvs produced by the *pen-huscfv*-transformed BL21 (DE3) *E. coli.* Lane M, protein molecular weight markers. Lanes 1 and 2, PEN-HuscFv27; lanes 3 and 4, PEN-HuscFv33; and lanes 5 and 6, PEN-HuscFv34. Numbers at the left are protein masses in kDa. (**C**–**E**) PEN-HuscFv27, PEN-HuscFv33 and PEN-HuscFv34, respectively, purified from homogenates of respective *pen-huscfv-*pET23b+ transformed BL21 (DE3) *E. coli* clones by using TALON affinity resin and the resin-bound antibodies were eluted from the resin by 250 mM imidazole solution into fractions. Lanes M, protein standard marker; lanes 1, crude *E. coli* lysates; lanes 2, flow through fractions; lanes 3, wash fractions; lanes 4–8, fractions eluted with 250 mM imidazole solution; and lanes 9, 1 M imidazole eluted fractions. Numbers at the left are protein masses in kDa.

The HuscFvs were linked molecularly to a cell-penetrating peptide (CPP), penetratin (PEN), to make them cell-penetrable (became superantibodies). The *huscfvs* of HB2151 *E. coli* clones 27, 33 and 34 were subcloned from the respective *huscfv*-phagemids into *pen*-pET23b+ (*huscfv* was inserted downstream of the gene coding for PEN) and the recombinant DNA constructs were transformed into DH5α *E. coli*. Figure 5A shows results of direct colony PCR for amplification of *pen*-*huscfvs* from various *pen*-*huscfv*-*pET23b+* transformed DH5α *E. coli.* The *pen*-*huscfv-*pET23b+ were subcloned from DH5α *E. coli* to BL21 (DE3) *E. coli*. The selected transformed BL21 (DE3) *E. coli* clones were grown under IPTG induction condition for production of the respective PEN-HuscFvs in large scale (Figure 5B). The PEN-HuscFvs were purified from the transformed BL21 (DE3) *E. coli* homogenates using TALON affinity resin (Figure 5C–E).

### 2.6. Inhibition of 3CL^pro^ Enzymatic Activity by PEN-HuscFvs 

**Figure 6:**
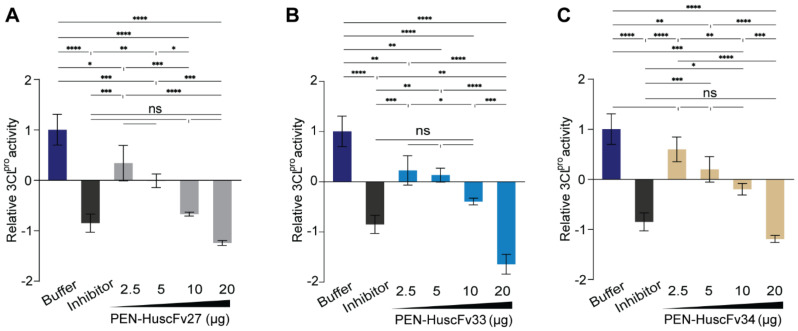
Inhibition of 3CL^pro^ activity by the PEN-HuscFvs as determined by FRET assay. In the assay, 20 μg of recombinant 3CL^pro^ was mixed with buffer (negative inhibition control), inhibitor (positive inhibition control), or various amounts of PEN-HuscFvs (2.5, 5, 10 and 20 μg). Individual mixtures were added to appropriate wells of a 96-well flat and clear bottom black plate and the plate was kept on an orbital shaker at 25 °C for 30 min. The r3CL^pro^ fluorescent substrate (5 μg) was added to each well and the plate was kept shaking for 4 h. Fluorescent signals were recorded at the excitation wavelength 436 nm and emission wavelength 528 nm using Synergy H1 Microplate reader. (**A**–**C**) are relative 3CL^pro^ activities in the presence of PEN-HuscFv27, PEN-HuscFv33, and PEN-HuscFv34, respectively. ns, not significantly different; * *p* ≤ 0.05; ** *p* ≤ 0.01; *** *p* ≤ 0.001; **** *p* ≤ 0.0001.

PEN-HuscFv27, PEN-HuscFv33 and PEN-HuscFv34 were tested for their ability to inhibit r3CL^pro^ activity in vitro. In this experiment, 20 µg of r3CL^pro^ was mixed with different amounts of PEN-HuscFvs before adding 5 µg of fluorogenic substrate and the preparations were kept at 25 °C for 4 h. Positive inhibition control (r3CL^pro^ mixed with protease inhibitor and substrate) and negative inhibition control (r3CL^pro^ mixed with buffer and substrate) were included in the experiments. The relative fluorescence units (RFU) at 528 nm were recorded and the 3CL^pro^ activities in the presence of PEN-HuscFvs/inhibitor in relation to the activity of negative inhibition control were calculated. The PEN-HuscFvs could inhibit the activity of the 3CL^pro^ in a dose-dependent manner, i.e., the 3CL^pro^ activities after treatment with 20 μg of PEN-HuscFvs were significantly less (*p* < 0.001) than treatment with 2.5–10 μg of the PEN-HuscFvs (Figure 6).

### 2.7. Biocompatibility of the PEN-HuscFvs to Human Cells

**Figure 7:**
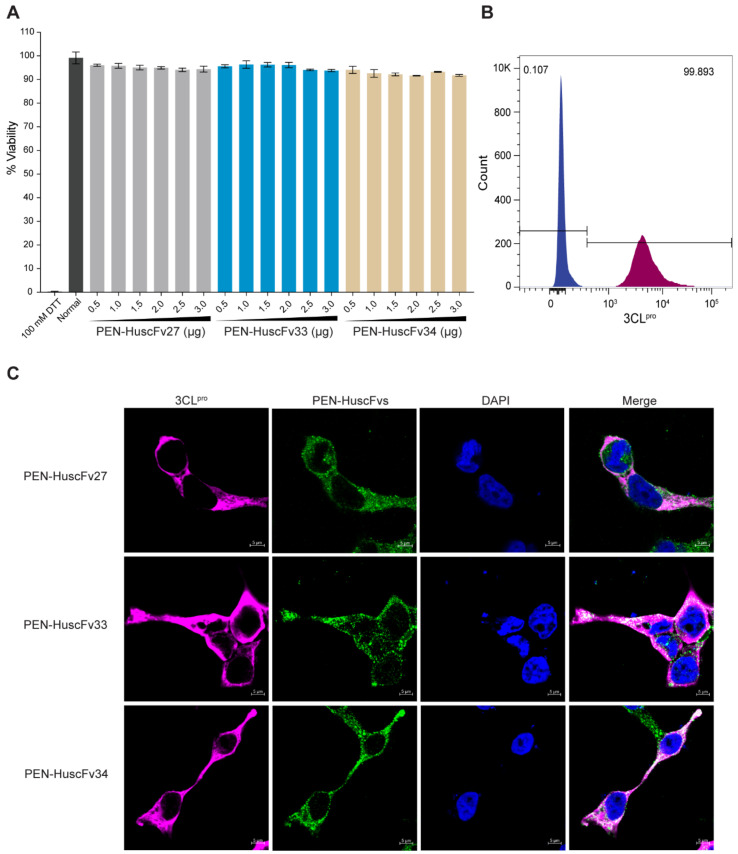
Biocompatibility of the PEN-HuscFvs to mammalian cells, mammalian cells expressing 3CL^pro^, and ability of the PEN-HuscFvs to enter mammalian cells. (**A**) Percent viability of human lung epithelial (A549) cells after exposure to 0.5–3.0 µM of PEN-HuscFv27, PEN-HuscFv33, and PEN-HuscFv34, compared to cells incubated with 100 mM DTT (cytotoxic control) and cells in culture medium alone (non-cytotoxic control; Normal). (**B**) Flow cytometric analysis of cells that expressed 3CL^pro^ (3CL^pro^-HEK293T cells; dark magenta) compared to cells without 3CL^pro^ (blue). (**C**) The 3CL^pro^-HEK293T cells containing intracellular 3CL^pro^ were incubated with PEN-HuscFv27, PEN-HuscFv33, and PEN-HuscFv34. The PEN-HuscFvs (green) were found to co-localize with the intracellular 3CL^pro^ (magenta) which then appear light pink or white upon merging. Nuclei stained blue by DAPI.

Human lung epithelial cells (A549) were incubated with 0.5–3.0 µM of PEN-HuscFv27, PEN-HuscFv33, PEN-HuscFv34, compared to cells incubated with 100 mM DTT (cytotoxic control) and cells in culture medium alone (non-cytotoxic control; Normal). The PEN-HuscFvs did not cause cytotoxicity to the human cells as measured using the CytoTox-Glo™ Cytotoxicity Assay, based on the Practical Guide to ISO 109903-5 [34] (Figure 7A). The PEN-HuscFv-treated cells appeared unchanged in their morphology (size and shape) under the light microscopy (data not shown).

### 2.8. Mammalian Cells Expressing Intracellular 3CL^pro^

HEK293T cells that stably express r3CL^pro^ were prepared by transfecting *3cl^pro^*-pTriEx1.1 plasmid into HEK293T cells and the cells that carried the *3cl^pro^*-pTriEx1.1 plasmid were selected by using hygromycin B drug. As shown in Figure 7B, flow cytometric analysis revealed that 99.8% of the transfected cells that survived the hygromycin B selection could produce intracellular 3CL^pro^ (designated 3CL^pro^-HEK293T cells) compared to the non-transfected HEK293T cells.

### 2.9. Cell-Penetrating Ability of the PEN-HuscFvs

The PEN-HuscFv27, PEN-HuscFv33 and PEN-HuscFv34 were tested for their cell-penetrating ability by incubating them with the 3CL^pro^-HEK293T cells. The 3CL^pro^-HEK293T cells incubated with the PEN-HuscFvs were washed, fixed, permeated and stained with a rabbit anti-HA tag antibody for detecting the 3CL^pro^, and mouse anti-6× His tag antibody for detecting the HuscFvs. Goat anti-rabbit Ig-AlexaFluor555 and goat anti-mouse Ig-AlexaFluor488 conjugate were used as secondary antibodies, while DAPI was used to locate nuclei. After washing, the cells were mounted on glass slides and observed under a confocal microscopy. As shown in Figure 7C, the PEN-HuscFvs (green) could enter the mammalian cells (being cell-penetrable antibodies/superantibodies), and co-localized with the intracellular 3CL^pro^ (magenta) which then appear light pink/white in merge panels.

### 2.10. Cell-Penetrable PEN-HuscFvs (Superantibodies) Inhibited Replication of SARS-CoV-2 Wild Type and Variants of Concern (VOC)

**Figure 8:**
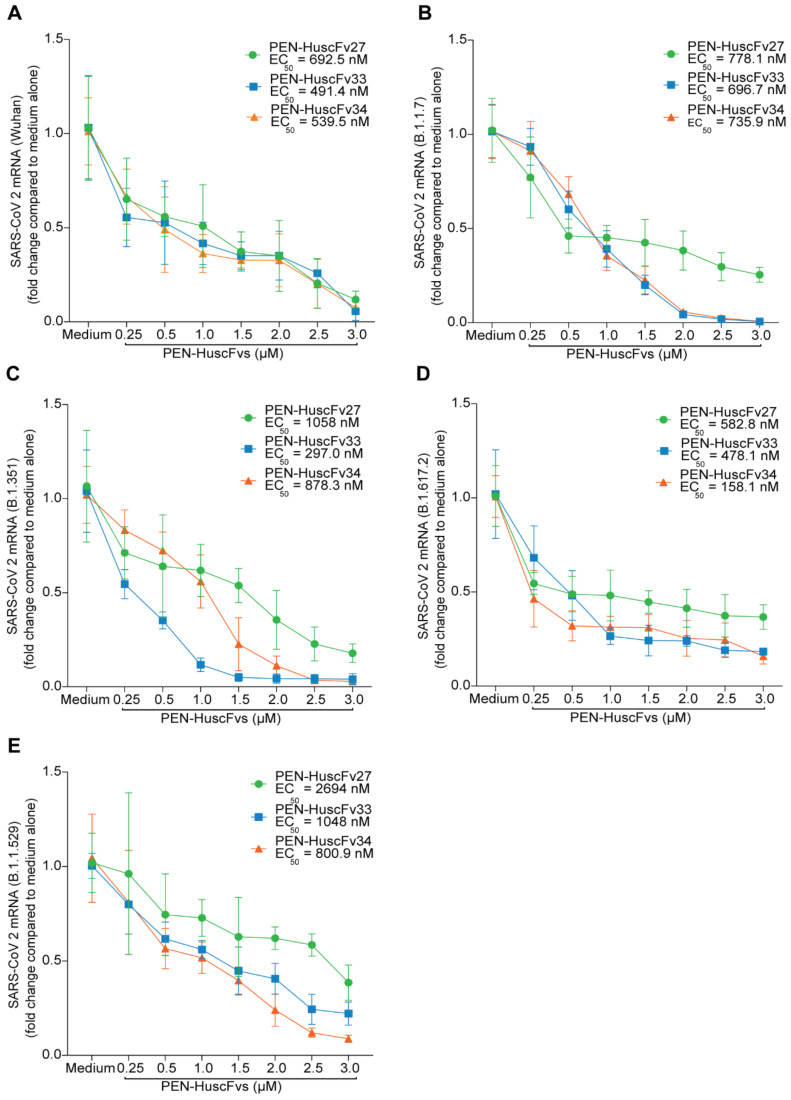
The anti-SARS-CoV-2 activity of the PEN-HuscFvs (superantibodies) in reduction of intracellular viral mRNAs. SARS-CoV-2 mRNA in infected cells treated with different concentrations of the PEN-HuscFv27, PEN-HuscFv33, and PEN-HuscFv34 compared to infected cells exposed to medium alone. The half-maximal effective concentrations (EC_50_) of individual superantibodies against indicated SARS-CoV-2 wild type and variants are shown in individual Figure insets. (**A**) Wuhan wild type; (**B**) α (B.1.1.7); (**C**) β (B.1.351); (**D**) δ (B.1.617.2) and (**E**) Omicron (B.1.1.529) variants.

**Figure 9:**
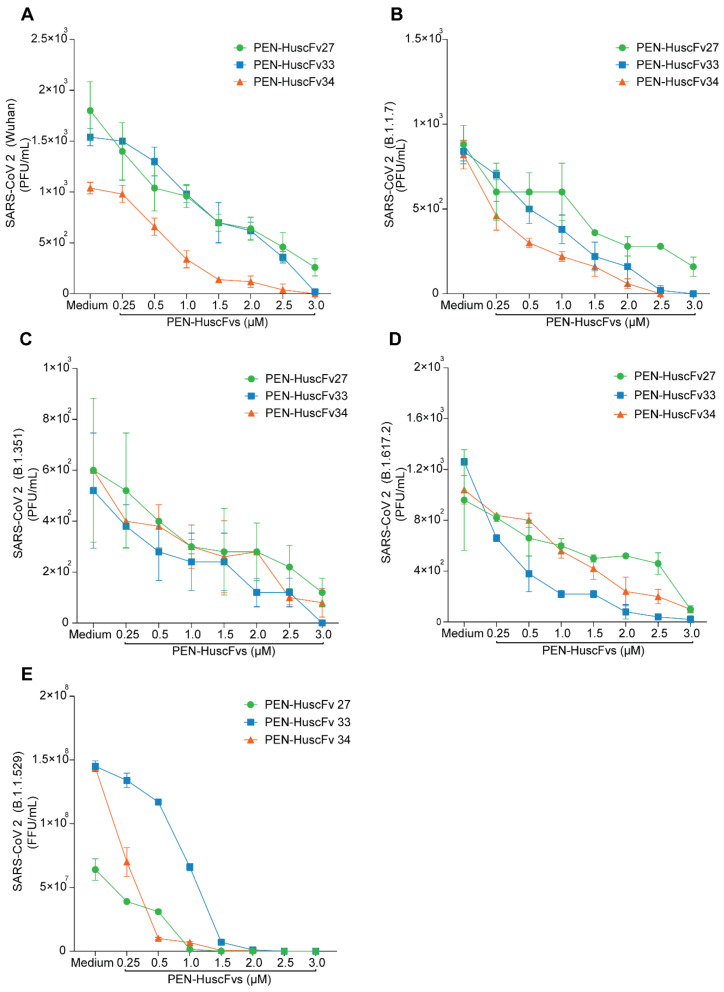
The anti-SARS-CoV-2 activity of the PEN-HuscFvs (superantibodies) in reducing the numbers of infectious virions (PFU/mL or FFU/mL) released from infected cells. The numbers of the infectious virions were determined by plaque forming assay (PFA) for Wuhan wild type and α, β and δ variants, and by focal formation assay (FFA) for Omicron variant. (**A**) Wuhan wild type; (**B**) α (B.1.1.7); (**C**) β (B.1.351); (**D**) δ (B.1.617.2) and (**E**) Omicron, B.1.1.529 variants.

The PEN-HuscFvs (superantibodies) were tested for replication inhibition of SARS-CoV-2 including Wuhan wild type and various mutated descendants (variants of concern; VOC) based on the previously described protocol [35]. Vero cells were pre-treated with different concentrations of the cell-penetrable PEN-HuscFvs for 3 h and the culture supernatants were collected and kept aside. The cells were added with virus for 1 h. Extracellular viruses were removed, and the kept culture supernatants were added back to the infected cells in the respective wells. After 18 h, virus RNAs in the infected cells were determined by qRT-PCR while the infectious virus particles in the culture supernatants were titrated by plaque forming assay (PFA) for Wuhan, α (B.1.1.7), β (B.1.351), and δ (B.1.617.2) variants and by focal forming assay (FFA) for Omicron (B.1.1.529) variant. The results of the qRT-PCR and the plaque/focal formation assays are shown in Figure 8 and Figure 9, respectively. The superantibodies readily inhibited replication of all SARS-CoV-2, including the Wuhan wild type and the mutated variants in the antibody-dose dependent conduct. Half-maximal effective concentration (EC_50_) of the PEN-HuscFv27, PEN-HuscFv33 and PEN-HuscFv34 are shown in Table 2 and insets of Figure 8.

## 3. Discussion

Coronaviruses infect a broad range of vertebrate hosts including human and diverse animal species, i.e., domestic, wildlife, companion, rodents, and avian species, causing severe respiratory and/or digestive diseases. Due to rapid mutation rate and high ability to adapt to the host environment, coronaviruses render a constant threat of human and animals through unpredictable emergence of new strains/genetic variants and their cross-species transmissibility. During the past two decades, the world population has been threatened by three highly fatal zoonotic respiratory diseases, caused by coronaviruses of the genus *Betacocoravirus*, namely SARS-CoV in 2003, MERS-CoV in 2012, and SARS-CoV-2 in late 2019 and up to now. The most recent coronavirus disease 2019 (COVID-19) pandemic caused by the SARS-CoV-2 has infected 512.6 million people of which 6.243 million were deceased (reported by the World Health Organization; 14 April 2022) and inflicts severe civil and economic hazard worldwide. Currently, new SARS-CoV-2 variant of concern (VOC) with rapid spread and high infectiousness continues to infect more people, both unvaccinated and vaccinated subjects [36]. The optimistic scenario for COVID-19 is that the pandemic, sooner or later, will become an endemic, the same as influenza, i.e., that SARS-CoV-2 may turn to cause seasonal epidemics which require seasonal/annual immunization against the circulating virus variant, as well as specific therapeutic agent for preventing/mitigating symptom severity, especially in vulnerable subjects.

Antibodies have been used for treatment and intervention of human diseases, both non-infectious and infectious, including viral diseases [37,38,39]. Serum samples of vaccinated or COVID-19 convalescing subjects containing polyclonal neutralizing antibodies [40,41,42] can be used as a therapeutic option for COVID-19 [43]. The neutralizing polyclonal antibodies may interfere with the virus cellular entry via direct blocking of the virus ligand (RBD/RBM of S1 subunit), steric hindrance of virus–receptor interactions, and/or prevention of S2 subunit-mediated genome uncoating and release. Other mechanisms may involve complement-mediated virus/infected cell lysis, opsonization of the virus particles for enhanced phagocytosis and antibody-dependent cell-mediated cytotoxicity (ADCC) [42]. Limitations and obstacles of COVID-19 treatment using convalescent or vaccinated serum besides ethical issue and supply inadequacy, are adverse reactions associated with plasma transfusion ranging from fever to serious hazards which are life-threatening, including allergic reactions, bronchospasm, transfusion-related acute lung injury (TRALI), and circulatory overload (TACO)/hypervolemic, especially in patients with cardiorespiratory disorders [44,45]. There is also a risk of anthroponosis. The World Health Organization recommended the use of convalescent plasma only in severe and critical COVID-19 patients and against its use in non-severe COVID-19 patients [46]. Neutralizing monoclonal antibodies derived from a variety of sources including B cells of COVID-19 patients, immunized humanized (hACE2) mice, transgenic mice, and phage display library have been developed for COVID-19 treatment perspective (reviewed by Hwang, et al. [47]). Most of the monoclonal antibodies (human/humanized-/chimeric-) target spike protein, particularly the RBD or RBM; therefore, their therapeutic effectiveness can be varied due to the target mutations of the infecting/circulating virus variants. Several options to cope with neutralizing antibody escape SARS-CoV-2 mutants have been explored, e.g., the use of cocktail of two neutralizing antibodies specific to non-overlap epitopes of SARS-CoV-2 spike protein [48], engineered bivalent IgG, and synthetic tetravalent neutralizing antibodies [bivalent diabody-Fc (DFc) and bi-paratopic tetravalent diabody-Fc-Fab (D-Fc-F) formats] against SARS-CoV-2 [49] which formed multivalent interactions with a single S-protein trimer and blocked the virus entry. Other therapeutic monoclonal antibodies are directed at proinflammatory cytokines (IL-6, IL-1β, TNF and GM-CSF) to mitigate cytokine storm syndrome (CSS) that is the frequent cause of fatal acute respiratory distress syndromes (ARDS) in COVID-19 patients [47]. High affinity humanized monoclonal antibodies to complement products including C5, C5a and C5aR to block the activities of the anaphylatoxin and the formation of membrane attack complex (MAC) associated with ARDS in COVID-19 have been developed for reducing hypoxia and increasing survival in patients with severe COVID-19 [47,50]. The issue of high concern in the treatment of viral diseases using intact four-chain antibodies, either monoclonal or polyclonal, directed to the virus surface exposed components is the antibody-dependent enhancement (ADE). For respiratory virus infections, the exacerbated diseases caused by ADE is often observed after vaccination, i.e., vaccine associated-enhanced respiratory disease (ERD) [51]. Different types of ADE have been recognized in coronavirus infections. The immune complexes formed between the antibodies (passively transferred or vaccine-induced) and the viruses cause exacerbated inflammation through complement activation and recruitment of inflammatory and immune cells, resulting in vital organ damage; designated immune enhancement ADE [52]. The intracellular virus may stimulate Th2 type immune response via increment of suppressor of cytokine proteins (SOCs) and inhibition of innate anti-viral immunity, hence heightening the virus production (intrinsic ADE) [53]. The antibody promotes up/open form of the RBD which enhances the coronavirus entry into host cells [35]. The antibodies may enhance the entry of viruses into monocytes/macrophages via the Fc receptors. Although in the case of the SARS-CoV-2, the macrophage/monocyte infection is abortive [54]; nevertheless, the virus induces a specific M2 macrophage transcriptional program and causes host immune paralysis for the benefit of COVID-19 progression and pathogenesis [54].

Chymotrypsin-like cysteine protease (3CL^pro^) is an attractive therapeutic target of an anti-coronavirus agent for many reasons. The enzyme is highly conserved and indispensable for coronavirus life cycle [55]. It plays a pivotal role in processing the virus polyproteins into mature non-structural proteins (nsps) with different functions during the early stage of the virus replication cycle. Thus, inhibition of the 3CL^pro^ would eventually prevent the infected cells from producing new infectious virions. Most of all, there is no human homolog of the 3CL^pro^, which means that targeting 3CL^pro^ should be a relatively safe endeavor. Several molecular inhibitors that block the 3CL^pro^ have been screened and tested [26,29,56,57]. The small molecular inhibitors could traverse across the otherwise formidable plasma membranes, access the cytosolic 3CL^pro^ and inhibit the protease activity therein [58]. However, a major limitation of these inhibitors is their off-target toxicity, which hinders patients’ compliance in clinical applications [56,59]. Besides, drug-escape virus mutants emerge rather easily [60,61]. In this study, another therapeutic option for coronavirus disease is offered, i.e., the non-neutralizing but cell-penetrable antibodies that bind to intracellular 3CL^pro^ and inhibit the enzyme activity leading to interference with the virus replication cycle. To our knowledge, this is the first study on cell-penetrating fully human antibodies that target the cytosolic 3CL^pro^ of coronavirus. The strategy of using cell-penetrable human antibodies to intracellular cellular viral proteins should be a new therapeutic paradigm for the treatment of virus infections. Unlike neutralizing antibodies that inhibit virus entry, the cell-penetrating antibodies allow the virus to enter but then inhibit the replication and progeny production. The antibodies that can bind to target inside the cells are called “Super-antibodies” (the term coined by Charles Morgan, president of InNexus Biotechnology, Vancouver, BC, Canada). The superantibodies bind to the intracellular target; if there is no target, they leave the cell and enter new cells. “In theory, they could do everything that the small molecules of most conventional drugs do, and more. The beauty of a cell-penetrating superantibody is that it would be highly discriminating. Because antibodies can be far more specific than small-molecule drugs, and because they are not inherently toxic, they should have fewer side effects” [62].

In this study, the engineered fully human single-chain antibody variable fragments (VH-linker-VL; HuscFvs) to 3CL^pro^ were generated using phage display technology [32,63]. Each HuscFv molecule contains six complementarity determining regions (CDRs), i.e., three CDRs each in the VH and the VL, that are configured by canonical framework regions (FRs). Within individual CDRs, several amino acid residues work synergistically in creating binding specificity to different sites of the target. Thus, not only the HuscFvs create a high binding affinity (nanomolar range, similar to the chemical drugs; besides, the antibody affinity can be increased by CDR resurfacing/point mutations), but they also render the relative difficulty for the virus to develop the antibody escape-3CL^pro^ mutant that retains the inherent 3CL^pro^ protease activity. For safety issue, therapeutic antibodies should have negligible or no immunogenicity in the recipients, implying that the small fully human antibody fragments (with high tissue penetrating ability and robustness) should be the safest isotype and effective format for human disease treatment. Being derived from the human’s own genes contained in the 3CL^pro^-bound phages, the HuscFvs to 3CL^pro^ produced by *E. coli* infected with the 3CL^pro^-bound phages in this study should not be immunogenic when used to treat patients with coronavirus infections (safe). The HuscFvs are devoid of Fc fragments and cannot form large immune complexes, and target intracellular protein; thus, they should not cause the detrimental ADE that is frequently observed when intact antibodies targeting the virus surface protein were used in treatment.

The bacterially derived recombinant 3CL^pro^ with specific protease activity (as tested by the FRET assay) was used in the phage panning such that some of the antigen-bound phages would bind to the critical regions and residues of the correctly folded recombinant 3CL^pro^. The virally produced 3CL^pro^ was not used as it is arduous to obtain the naïve enzyme in adequate quantity. Single-round phage-panning was performed instead of multiple-round panning used by other laboratories; because our experience with this self-constructed HuscFv phage display library [32,64] indicated that multiple panning rounds tended to result in the loss of HuscFv genes from the phages. The phage library was subtracted with lysate of original BL21 (DE3) *E. coli*, the expression host of r3CL^pro^, before incubating with the r3CL^pro^ to minimize non-specific binding phages. Additionally, the phage transfected *E. coli* clones were screened for not only those that harbored recombinant *huscfv*-phagemids, but also thosethat could express HuscFvs that bound to the panning 3CL^pro^ as well as the 3CL^pro^ produced by transfected mammalian cells (the 3CL^pro^ should acquire natural fold). Because there were many *E. coli* clones that expressed HuscFvs bound to the r3CL^pro^ by the indirect ELISA, the computerized simulation was used to guide the selection of only the clones that their HuscFvs formed contact interfaces with critical parts of the 3CL^pro^ that participate in active protease activity, including the residues that form catalytic diad, substrate binding residues, and/or residues involved in homodimerization for the formation of the active enzymatic module. From the in-silico selection, three transformed *E. coli* clones (27, 33 and 34) were produced HuscFvs with the required target specificities. Therefore, the HuscFvs of these three *E. coli* clones were linked molecularly to a cell-penetrating peptide, penetratin (PEN) such that they become cell-penetrable and accessible to the intracellular 3CL^pro^, being super-antibodies. This strategy was used successfully in our laboratory for making cell-penetrable antibodies (superantibodies) to other intracellular targets, such as influenza matrix protein [65,66], proteins of hepatitis C virus, Ebola virus and HIV-1 [67,68,69,70,71,72,73,74,75]. The PEN-HuscFv27, PEN-HuscFv33 and PEN-HuscFv34 could readily enter human cells and bound to intracellular 3CL^pro^. They were not toxic to the human cells. The penetratin (PEN) is not immunogenic and is currently used as a vaccine delivery vehicle [76,77]. Most of all, the superantibodies inhibited replication of SARS-CoV-2 Wuhan wild type and the variants of concern with RBD mutations particularly the highly contagious delta variant with two point mutations (L452R and T478K) in the RBD [78] and the omicron variant with 15 mutated RBD residues (G339D, S371L, S373P, S375F, K417N, N440K, G446S, S477N, T478K, E484A, Q493K, G496S, Q498R, N501Y, and Y505H) [79]. Based-on the fact that 3CL^pro^ of the currently recognized betacoronaviruses are highly conserved at the protease critical residues, there is no reason that the PEN-HuscFvs of this study should not inhibit replication of the other SARS-CoV-2 descendants that may unprecedentedly emerge in the future (as the mutated residues that rendered them highly contagious tend to locate mainly in the spike protein), as well as the other betacoronaviruses. Unfortunately, other betacoronaviruses, e.g., SARS-CoV and MERS-CoV, are not available for testing the replication inhibitory activity of the super-antibodies. It would also be interesting to compare the SARS-CoV-2 inhibitory activity of the superantibodies to the 3CL^pro^ produced in this study with the anti-S antibodies [48,49] and their combined/synergistic effects as well as to demonstrate the effect on the expression profiles of genes and proteins involved in innate immunity and inflammation in Vero cells after treatment with the superantibodies [48].

In conclusion, human single-chain superantibodies that target SARS-CoV-2 3CL^pro^, the major protease that are shared by all betacoronaviruses and pivotal for the virus replication cycle, were generated in this study. These small antibody fragments should not have immunogenicity in the recipients as the HuscFvs were from human immunoglobulin genes and the penetratin which was used as the protein transduction domain is not immunogenic. In addition, they should not cause ADE as they target intracellular virus protein, lack Fc portion, cannot cross-link the target, cannot form large immune complex, and cannot activate complement. The superantibodies are not toxic to human cells. Most of all, they broadly and effectively block replication of SARS-CoV-2 wild type and the mutated VOC descendants. Thus, the superantibodies to the 3CL^pro^ should be tested further towards a clinical use against not only SARS-CoV-2, but also other betacoronaviruses.

## 4. Materials and Methods

### 4.1. Cell Lines, Cell Cultures and Virus Propagation

Human embryonic kidney cells (HEK293T), human lung epithelial cells (A549) and African green monkey kidney (Vero) cells were from American Type Culture Collection (ATCC, Manassas, VA, USA). Vero E6 cells (VERO C1008) were kindly provided by Prof. Dr. Prasert Auewarakul, Department of Microbiology, Faculty of Medicine Siriraj Hospital, Mahidol University, Bangkok. The cells were cultured in Dulbecco’s modified Eagle’s medium (DMEM) (Gibco, Thermo Fisher Scientific,) supplemented with 10% fetal bovine serum (FBS) (Sigma; Merck KGaA, Damstadt, Germany), 100 units/mL penicillin, 100 µg/mL streptomycin, and 2 mM L-glutamine (Gibco) (complete DMEM).

SARS-CoV-2 isolates used in this study included Wuhan wild type and α (B.1.1.7), β (B.1.351), δ (B.1.617.2) and Omicron (B1.1.529) variants. They were isolated from infected Thai patients. The viruses were propagated in Vero E6 cells. The cells (4.2 × 10^6^ cells) were seeded to T75 flask (Nunc, Thermo Fisher Scientific) and incubated at 37 °C in 5% CO_2_ atmosphere overnight. The culture fluid was removed; SARS CoV-2 at MOI 0.01 in 3 mL of plain DMEM was added to the cell monolayer, incubated at 37 °C in the 5% CO_2_ incubator for 1 h, then 15 mL of complete DMEM was added to the culture flask. The infected cells were incubated further for 3–5 days or until the maximal CPE was seen. The culture fluid was collected, centrifuged, and kept in small portions at −80 °C. The virus titers were determined by plaque or focal forming assay.

### 4.2. Production of Recombinant 3CL^pro^ (r3CL^pro^)

Nucleotide sequence coding for SARS-CoV-2 3CL^pro^ was retrieved from GenBank database (accession No. MN90894.3) and synthesized as an inserted DNA in pET23a+ (Genscript, Piscataway, NJ, USA). The synthesized plasmid was used for preparing r3CL^pro^. DNA fragments coding for the 3CL^pro^ were excised from the *3cl^pro^*-pET23a+ and cloned into pTriEx1.1 with either 8× histidine tag (used for recombinant 3CL^pro^ production) or with HA tag (used for preparing 3CL^pro^ stably expressed cells) via the *Xho*I and *Not*I endonuclease restriction sites. The recombinant plasmid was used to transform DH5α *E. coli* by heat-shock transformation. To produce DH5α *E. coli* competent cells, 100 µL of the *E. coli* overnight culture in Lennox broth was inoculated into 10 mL of fresh Lennox broth and incubated at 37 °C with 250 rpm shaking until OD 600 nm was 0.5. The culture was centrifuged (4000× *g*, 4 °C, 15 min), and the pellet was resuspended with 5 mL of 100 mM ice-cold MgCl_2_. The suspension was kept in an ice-bath for 5 min. The pellet was collected after centrifugation (4000× *g*, 4 °C, 15 min), and resuspended with 1 mL of 100 mM ice-cold CaCl_2_. The suspension was chilled in an ice-bath at least 1 h. The competent cells were kept in small portions at −80 °C. For heat-shock transformation, the recombinant plasmid was mixed with 100 µL of the DH5α *E. coli* competent cells and chilled in an ice-bath for 20 min. The mixture was immediately heated in a 42 °C water-bath for 2 min and immediately chilled in an ice-bath for 5 min. Nine hundred microliters of LB broth was added and the culture was incubated at 37 °C with 250 rpm shaking for 1 h. The culture was centrifuged (4000× *g*, 4 °C, 15 min) and resuspended with 100 µL of Lennox broth The transformed preparation was spread onto a Lennox agar plate containing 100 µg/mL ampicillin and incubated at 37 °C overnight. *E. coli* colonies containing *3cl^pro^-*pTriEx1.1 plasmids were screened by direct colony polymerase chain reaction (PCR) (Tables S1 and S2) using T7 promoter and T7 terminator oligonucleotide primers (Table S3). The *3cl^pro^*-positive clone was grown, and the plasmids were extracted for Sanger sequencing (1st BASE, Seri Kembangan, Selangor, Malaysia). The verified recombinant plasmids were transformed into NiCo21 (DE3) *E. coli* expression host as described above. One of the transformed NiCo21 (DE3) *E. coli* colonies with recombinant *3cl^pro^-*pTriEx1.1 plasmid was grown in Lennox broth containing 100 µg/mL ampicillin at 37 °C with shaking aeration (250 rpm) overnight. One hundred microliters of the overnight culture was added to 10 mL of fresh Lennox broth containing 100 µg/mL ampicillin and incubated with 250 rpm shaking until the OD at 600 nm reached 0.5. Isopropyl β-d-1-thiogalactopyranoside (IPTG) was added to the culture (0.1 mM final concentration) and the culture was incubated further for 3 h. The bacterial cells collected from the culture after centrifugation (4500× *g*, 4 °C, 15 min) were resuspended in 1 mL phosphate buffered saline, pH 7.4 (PBS) and homogenized by sonication (30% amplitude) in an ice bath for 1 min. The supernatant collected after centrifugation (12,000× *g*, 4 °C, 20 min) was checked for the presence of the 6× His tagged-recombinant protein by Western blot analysis.

For the Western blot analysis, the antigen (supernatant/bacteria lysate) was mixed with 6× sample buffer containing sodium dodecyl sulfate (SDS) and 2-mercaptoethanol and boiled for 5 min before being subjected to sodium dodecyl sulfate-polyacrylamide gel electrophoresis (SDS-PAGE; 4% stacking gel and 12% separating gel) cast in Mini-PROTEAN^®^ Tetra Handcast Systems (Bio-Rad, Hercules, CA, USA). The separated components were transblotted onto a nitrocellulose membrane (NC). After blocking the unoccupied sites on the blotted NC with 5% skimmed milk for 1 h and washed thrice with Tris buffered saline solution containing 0.05% Tween-20 (TBT-T), the NC was placed in a solution of mouse anti-6× His monoclonal antibody (Bio-RAD) and kept at room temperature (25 ± 2 °C) for 1 h. After three washes with TBS-T, the NC was incubated with goat-anti-mouse Ig-alkaline phosphatase (AP) conjugate (SouthernBiotech, Birmingham, AL, USA) for 1 h, washed as above, placed in KPL BCIP/NBT substrate solution (SeraCare, Milford, MA, USA) and kept in darkness. The enzymatic reaction was stopped by rinsing the NC with distilled water when the antigen-antibody reactive bands were visualized. The 6× His tagged-r3CL^pro^ in the *E. coli* lysate was purified by using TALON^®^ Metal Affinity resin [Clontech Lab, CA, USA (presently Takara Bio USA)] and eluted by 150 mM imidazole solution into 1 mL-fractions.

### 4.3. Fluorescence Resonance Energy Transfer (FRET) Assay for Determining 3CL^pro^ Enzymatic Activity and 3CL^pro^ Inhibition Assay

In this study, a recombinant fusion protein consisted of cyan fluorescent protein (CFP) linked to 3CL^pro^ substrate peptide (TSAVLQSGFRKM) and yellow fluorescent protein (YFP): CFP-TSAVLQSGFRKM-YEP was produced and used as the 3CL^pro^ substrate in the FRET assay. The gene coding for the 6× His-CFP-TSAVLQSGFRKM-YFP fusion protein was synthesized and inserted to a pQE-1 plasmid vector (Genscript). The plasmid was transformed to *E. coli* strain M15 by heat shock transformation as described above. The preparation was spread the Lennox agar plate containing 100 µg/mL ampicillin and 50 µg/mL kanamycin and incubated at 37 °C overnight. A colony of transformed M15 *E. coli* harboring the plasmid that grew on the plate was grown in Lennox broth containing 100 µg/mL ampicillin and 50 µg/mL kanamycin at 37 °C with shaking aeration (250 rpm) overnight. The overnight culture was seeded to 50 mL fresh Lennox broth containing 100 µg/mL ampicillin and 50 µg/mL kanamycin in 250 mL-Erlenmeyer-flask to 1% final concentration and cultured at 37 °C with shaking aeration until OD 600 nm reached 0.3. Isopropyl β-d-1-thiogalactopyranoside was added to the culture (1 mM final concentration) and the culture was grown further for 3 h. The bacterial cells collected from the culture after centrifugation (4500× *g*, 4 °C, 15 min) were resuspended in purification buffer (50 mM phosphate buffer, 300 mM NaCl, pH 8.0) and homogenized by sonication (35% amplitude, 2 s on/off cycle) in ice-bath for 10 min. The supernatant collected after centrifugation (10,000× *g*, 4 °C, 15 min) containing 6× His-CFP-TSAVLQSGFRKM-YFP fusion protein (shown by yellow color) was added to TALON affinity resin (Clontech) preconditioned with native purification buffer and slowly rocked at 4 °C for 3 h. The mixture was loaded on an empty PD-10 column (Merck KGaA, Darmstadt, Germany); the resin was allowed to set by gravitation, then the flow-through fraction was released from the column, and the resin was washed by five column-volumes of the purification buffer. The 6× His-CFP-TSAVLQSGFRKM-YFP fusion protein was eluted from the column by adding 1 mL of 250 mM imidazole in native purification buffer, retained for 2 min, and collected from the column. The elution was repeated four more times (five eluted fractions were collected). The eluted fractions were checked for the presence of the 6× His-CFP-TSAVLQSGFRKM-YFP fusion protein and the protein purity by SDS-PAGE and Coomassie Brilliant Blue G-250 (CBB) staining and Western blotting. The 3CL^pro^ substrate protein was dialyzed against 20 mM Tris, pH 7.4, and filtered through 0.2 µM syringe filter (Acrodisc^®^, Pall Medical, Fribourg, Switzerland) for use in the FRET assay.

For the FRET assay, 80 μg of r3CL^pro^ was mixed with 5 μg of the substrate and kept at 25 °C for 60 min. The enzymatic activity was measured at 5-min intervals by using a Synergy H1 microplate reader (Biotek, Santa Clara, CA, USA) at an excitation wavelength of 436 nm and an emission wavelength of 528 nm. Alternatively, varying amounts (0.625–80 μg) of r3CL^pro^ samples were mixed individually with 5 μg of the fluorogenic substrate and kept at 25 °C for 4 h. The r3CL^pro^ enzymatic activity was measured.

### 4.4. HuscFv Phage Display Library

The phage library displaying human single-chain antibody variable fragments (HuscFvs) used in this study was constructed previously [32,64]. Briefly, complementary DNA reversely transcribed from a pool of total RNA extracted from the peripheral blood mononuclear cells (PBMCs) of 60 healthy donors was used as a template for PCR amplification of genes coding for all families and subfamilies of human Ig variable fragments (VH and VL domains). The primers used in the PCR were 48 pairs of degenerate oligonucleotide primers for human *vh* sequences (16 forward and three reverse primers) and 26 pairs of degenerate primers for human *vl* sequences (13 forward and two reverse primers) [32]. The amplified *vh* and *vl* sequences were linked randomly [via a polynucleotide linker coding for (Gly_4_Ser_1_)3] to yield a repertoire of gene sequences coding for human single-chain antibody variable fragments (*vh*-linker *vl* or *huscfvs).* The *huscfv* repertoire was cloned into pCANTAB 5E phagemids downstream of the phage *gIII* (coding for phage coat protein p3). The recombinant phagemids were introduced into competent TG1 *E. coli*. The phagemid-transformed TG1 *E. coli* bacteria were grown and co-infected with helper phage, M13KO7. The complete phage particles displaying the HuscFvs as fusion partners of the phage p3 protein on the surface of phage particles and contained the respective HuscFv genes (*huscfvs*) in the phage genomes were recovered from the *E. coli* culture supernatant. The HuscFv phage display library was obtained.

### 4.5. Production of HuscFvs to r3CL^pro^

Human single-chain antibody variable fragments (HuscFvs) to r3CL^pro^ were produced by using phage display technology as described previously [32]. The enzymatically active r3CL^pro^ was used as an antigen in the phage bio-panning to select out the HuscFv-displaying phage clones from the HuscFv phage display library. In the phage bio-panning, 10 µg of the r3CL^pro^ and lysate of original BL21(DE3) *E. coli* (expression host of r3CL^pro^), in 100 µL of PBS, were added to different wells of EIA/RIA strip (Corning, Thermo Fisher Scientific) and kept at 4 °C overnight. The wells were washed with PBS containing 0.05% Tween-20 (PBS-T); blocked with 3% bovine serum albumin (BSA) in PBS (300 µL) and kept at 37 °C for 1 h. After removing the excess BSA by washing with the PBS-T, 50 µL of the HuscFv phage display library (~10^11^ phage particles) was added to the well coated with the original HB2151 *E. coli* lysate. The plate was kept at 37 °C for 1 h, then the fluid containing the unbound phages (subtracted library) was transferred to the r3CL^pro^ coated well and incubated at 37 °C for 1 h. The fluid containing unbound phages was discarded; the well was washed 10 times with PBS-T (300 µL each time). Log phase grown HB2151 *E. coli* (100 µL) was added to the well containing the r3CL^pro^-bound phages and kept for 15 min to allow phage infection of the bacteria. The fluid was collected, and another 100-µL aliquot of the HB2151 *E. coli* was added to the well. After 15 min, the fluid was collected and combined with the first fluid aliquot. The fluid pool was diluted 10-fold serially and each dilution was spread onto 2× YT-ampicillin agar plates containing 2% glucose (2YT-AG agar) and the plates were incubated at 37 °C overnight.

The phagemid transformed-HB2151 *E. coli* clones that grew on the agar were screened for *huscfvs* by direct colony PCR using R1 forward primer specific to the pCANTAB 5E phagemid (Tables S1–S3). The HB2151 *E. coli* clones positive for the *huscfvs* were grown in 2× YT-AG broth at 37 °C with shaking aeration (250 rpm) until the OD 600 nm were 0.3–0.5. The bacterial cells were collected after centrifugation of the cultures, resuspended in fresh 2× YT-A broth containing 1 mM IPTG, and incubated further for 3 h. The cultures were centrifuged to collect the bacterial pellets, which were then resuspended individually in 1 mL PBS. The bacterial cells were sonicated, and the homogenates were centrifuged (12,000× g, 4 °C, 20 min). The bacterial lysates were collected and the presence of E-tagged HuscFvs was determined by Western blot analysis using mouse anti-E tag antibody to probe the HuscFvs in the SDS-PAGE-separated lysates.

### 4.6. Indirect Enzyme-Linked Immunosorbent Assay (Indirect ELISA)

Soluble HuscFvs in the lysates of HB2151 *E. coli* were tested for binding to r3CL^pro^ by indirect ELISA using BSA as the control antigen [64]. For the indirect ELISA, r3CL^pro^ and BSA (100 µg in 100 µL PBS) were added separately to wells of a 96-well-ELISA plate and kept at 4 °C overnight. After washing with Tris buffered saline containing 0.1% (*v*/*v*) Tween-20 (TBS-T) and blocking with 5% (*w*/*v*) skimmed milk, 100 µL of individual *E. coli* lysates were added to antigen and control antigen coated wells for 1 h. After washing with TBS-T, wells were added with rabbit anti-E tag (1:3000 dilution, ab3397, Abcam, Cambridge, United Kingdom) to detect HuscFvs, for 1 h. The signal was developed by adding 1:3000 diluted horseradish peroxidase (HRP)-conjugated goat anti-rabbit isotype (SouthernBiotech) for 1 h, followed by KPL ABTS substrate (Sera Care) for 30 min with TBS-T washing three times between the steps. The HB2151 *E. coli* clones that the HuscFvs in their lysates gave OD 405 nm to r3CL^pro^ at least two times higher than the same lysate to the control antigen were selected for further experiments.

### 4.7. Determination of Nucleotide and Amino Acid Sequences, Complementarity Determining Regions (CDRs) and Immunoglobulin Framework Regions (FRs) of the r3CL^pro^ Bound HuscFvs

The selected *E. coli* clones were grown in LB-A broth at 37 °C with shaking (250 rpm) overnight. The *huscfv*-phagemids they carried were isolated using the Presto™ mini plasmid kit (Favogen, Ping-Tung, Taiwan) and the *huscfvs* were Sanger sequenced (1st BASE). The verified nucleotide sequences were deduced to amino acid sequences using the CLC main work bench 8 (Qiagen, Hilden, Germany). Complementarity determining regions (CDRs) and immunoglobulin framework regions (FRs) of the amino acid sequences from individual *E. coli* clones were analyzed by using IMG/V-QUEST analysis tool and they were compared by Clustal Omega (https://www.ebi.ac.uk/Tools/msa/clustalo/; accessed on 30 December 2020).

### 4.8. Computerized Homology Modeling and Intermolecular Docking for Determining Presumptive Residues and Domains of 3CL^pro^ Bound by the HuscFvs

Computerized simulation was used for guiding selection of the HuscFv-expressing *E. coli* clones that their expressed HuscFvs formed contact interface with important residues of the 3CL^pro^. Three dimensional (3D) structures of HuscFvs from *E. coli* clones of interest were built through submission of the HuscFv amino acid sequences to RaptorX server (http://raptorx.uchicago.edu; accessed on 24 March 2021) for homology modeling. The 3D structure of SARS-CoV-2 3CL^pro^ was retrieved from http://zhanglab.ccmb.med.umich.edu/COVID-19/ (accessed on 15 February 2021). The 3D structures of HuscFvs and 3CL^pro^ were subjected to intermolecular docking using HADDOCK 2.4 webserver (https://wenmr.science.uu.nl/haddock2.4/; accessed on 15 March 2021). The protein-antibody complexes, which showed the largest clusters with the lowest interactive energy by prodigy webserver (https://wenmr.science.uu.nl/prodigy/; accessed on 25 March 2021) were selected. Residues and domains of the two parties that formed contact interface were analyzed using Discover Studio 2021 program.

### 4.9. Production of Cell-Penetrable Antibodies (Penetratin Linked-HuscFvs, PEN-HuscFvs)

The *huscfv* sequences of the selected *E. coli* clones were subcloned from phagemid vector to pET23b+ vector that already carried inserted gene sequence coding for penetratin (PEN), a cell-penetrating peptide (CPP; RQIKIWFQNRRMKWKK), i.e., *pen-pET23b+* vector [65]. The *huscfv-*pCANTAB 5E phagemids and the *pen*-pET23b+ vectors were digested doubly and similarly with *Sfi*I and *NotI* restriction endonucleases. The *huscfvs* were ligated to the cut *pen*-pET23b+ vectors and the recombinant plasmids were transformed into DH5α *E. coli* by heat-shock transformation as described previously. The transformed preparations were spread on LB-A agar plates and incubated at 37 °C overnight. The *E. coli* colonies grew on the plate and carried *PEN-HuscFv*-pET23b+ plasmids [screened by direct colony PCR using T7 promotor and terminator oligonucleotide primers (Tables S1–S3)] were grown in LB-A broth and the recombinant plasmids were extracted for verification by Sanger sequencing. The verified plasmids were transformed into BL21 (DE3) *E. coli* expression host by heat shock transformation as described above. The BL21 (DE3) *E. coli* clones with recombinant *PEN-HuscFv*-pET23b+ plasmids were detected by direct colony PCR using the T7 promotor and terminator oligonucleotide primers. Positive clones were grown in Lennox broth containing ampicillin (100 µg/mL) and incubated at 37 °C overnight. One hundred microliters of the overnight cultures were inoculated into 10 mL of fresh Lennox broth containing the ampicillin and grown at 37 °C with shaking (250 rpm) until OD 600 nm reached 0.3–0.5. Isopropyl β-d-1-thiogalactopyranoside was added to individual cultures (0.25 mM final concentration) and the preparations were incubated further for 3 h. The IPTG induced-bacterial cells were harvested by centrifugation (4000× *g*, 4 °C for 30 min); the bacterial pellet was resuspended in 500 µL of 50 mM sodium phosphate buffer, pH 7.0 containing 300 mM NaCl and 8 M urea (binding buffer), and boiled for 5 min. The preparations were centrifuged (12,000× *g*, 4 °C, 15 min); the supernatants were collected and mixed with 6× sample buffer containing SDS and 2-mercaptoethanol, boiled for 5 min, and subjected to SDS-PAGE (12% gel). The separated components were blotted onto NC, which was then blocked with 5% skimmed milk in Tris buffered saline, pH 7.6 containing 0.01% Tween-20 (TBS-T) for 1 h. The NC was washed with TBS-T three times before placing in a solution of mouse anti-6× His monoclonal antibody (Bio-RAD) and kept at 25 °C for 1 h, washed with TBS-T and submerged in a solution of goat-anti-mouse Ig-alkaline phosphatase conjugate (SouthernBiotech) for 1 h. The reactive bands were revealed by using BCIP/NBT substrate (KPL). The enzymatic reaction was stopped by rinsing the NC with distilled water.

For large-scale production of the recombinant PEN-HuscFvs, the selected BL21 (DE3) *E. coli* clones were cultured in 10 mL Lennox broth containing 100 μg/mL ampicillin at 37 °C, 250 rpm shaking, overnight. The overnight cultures were added separately to 250 mL of fresh Lennox broth containing 100 μg/mL ampicillin, incubated further until OD 600 nm was 0.3–0.5, then the cultures were induced by IPTG as above. The IPTG induced-bacterial cells were harvested by centrifugation (4000× *g*, 4 °C for 30 min), and the bacterial lysates were prepared in 20 mL of binding buffer. PEN-HuscFvs were purified from the *E. coli* lysates by using TALON affinity resin (Clontech) under denaturing condition and subjected to SADS-PAGE and CBB staining for recombinant protein band revelation.

### 4.10. Preparation of Mammalian Cells That Expressed Intracellular r3CL^pro^

Human embryonic kidney cells (HEK293T cells) were seeded into a well of a 24-well-culture plate (1.5 × 10^5^ cells/well) (Corning, NY, USA) and incubated at 37 °C in 5% CO_2_ incubator overnight. The transfection reagents, i.e., Lipofectamine™ 3000 transfection reagent (Catalogue number: 1IV09-L3000-008, Thermo Fisher Scientific) was prepared. In one test tube, 1 µL of P3000^TM^, 25 µL of Opti-MEM™I reduced serum, and 0.5 µg of *3cl^pro^*-pTriEx1.1 plasmid were added to the mix. In another test tube, 1.5 µL of Lipo3000 was mixed with 23.5 µL of Opti-MEM™ I reduced serum. Thereafter, contents of the two test tubes were mixed well, and kept at 25 °C for 15 min. The mixture was gently added dropwise into the established HEK293T in the well and the cells were incubated at 37 °C in humidified CO_2_ incubator for 72 h. Hygromycin B (InvivoGen, CA, USA; 500 µg/mL) was added to the cells and kept at 37 °C in 5% CO_2_ atmosphere for 5 days. The cells that contained the plasmids (hygromycin resistant cells) remained alive while the cells without the plasmid transfection were all dead. The hygromycin-resistant cells were tested for the presence of intracellular r3CL^pro^ by flow cytometric analysis.

For the flow cytometry, the hygromycin-resistant cells in 1.5-mL test tube were washed with PBS and fixed with 4% paraformaldehyde in PBS at 25 °C for 30 min. The fixed cells were washed three times with PBS and permeated with eBioscience™ Permeabilization Buffer (Cat. no.: 00-8333-56, Thermo Fisher Scientific) at 25 °C for 20 min. After washing with PBS, the cells were blocked with 10% human AB serum in PBS at 25 °C for 30 min, washed with PBS, incubated with rabbit anti-HA tag antibody (Abcam; ab137838) at 25 °C for 1 h and counterstained with goat anti-rabbit Ig-AlexaFluor647 conjugate (Cat. no.: A32733, Invitrogen, Thermo Fisher Scientific) at 25 °C in darkness for 1 h. The cells were washed with PBS and suspended in FACS buffer (2% fetal bovine serum, 0.02% sodium azide in PBS) before subjecting to flow cytometric analysis for intracellular 3CL^pro^ (BD LSRFortessa^TM^ Cell Analyzer, BD Biosciences, Franklin Lakes, NJ, USA).

### 4.11. Biocompatibility of the PEN-HuscFvs to Human Cells

Human adenocarcinomic alveolar basal epithelial cells (A549) were seeded into wells of 96-well-white plate and incubated at 37 °C in 5% CO_2_ atmosphere overnight. The cells were washed, added with various concentrations (0.5, 1.0, 1.5, 2.0, 2.5 and 3 µM) of PEN-HuscFvs from different *E. coli* clones, and incubated at 37 °C in 5% CO_2_ atmosphere overnight. The cytotoxicity of the PEN-HuscFvs to the A549 cells was determined using CytoTox-Glo™ Cytotoxicity Assay (Cat. No.: G9291, Promega, Madison, WI, USA). The assay buffer (50 µL) supplied with the test kit were added to individual wells containing the PEN-HuscFv-treated A549 cells. The positive cytotoxic control was the A549 cells treated with 100 mM of freshly prepared dithiothreitol (DTT), and the negative control was cells in culture medium alone (normal). The plate was placed on a slow orbital shaker at 25 °C for 15 min; then dead cell luminescence was detected by a using Synergy H1 microplate reader (Biotek). Thereafter, 50 µL of lysis reagent were added to all wells; the plate was placed on the shaker for 15 min further before total luminescence in each well was determined. Viable cell luminescence = Total luminescence–dead cell luminescence. Thereafter, percent viability was calculated: percent cell viability = (viable cell luminescence ÷ normal A549 cell luminescence) × 100.

### 4.12. Determination of Cell-Penetrating Ability of the PEN-HuscFvs

The HEK293T cells that expressed 3CL^pro^ (3CL^pro^-HEK293T cells) in 500 µL of complete DMEM containing 500 µg/mL of hygromycin B were seeded onto cover slips placed in wells of a 24-well-culture plate (1.5 × 10^5^ cells per slip) and incubated at 37 °C in 5% CO_2_ incubator overnight. The fluids were discarded and PEN-HuscFvs (2.5 μM) from individual *E. coli* clones were added to appropriate wells containing the established 3CL^pro^-HEK293T cells and kept in 37 °C in the 5% CO_2_ atmosphere overnight. The cells were washed with PBS; fixed and permeated with 500 µL of ice-cooled methanol:acetone on ice for 10 min. The cells were washed with PBS, blocked with 3% BSA in PBS at 25 °C for 30 min, washed with PBS, and incubated with rabbit anti-HA tag antibody (1:1000) (detect 3CL^pro^) and mouse anti-6× His tag antibody (1:1000) (detect HuscFvs) at 25 °C for 1 h. After washing with PBS, the cells were counterstained with goat anti-rabbit Ig-AlexaFluor555 (1:200) and goat anti-mouse Ig-AlexaFluor488 conjugate (1:200) on ice in darkness for 1 h. DAPI was used to locate nuclei. After washing, the cover slips were placed on glass slides and the cells were mounted and observed under a confocal microscopy.

### 4.13. HuscFv-Mediated Inhibition of the 3CL^pro^ Catalytic Activity

PEN-HuscFvs, 3CL^pro^ inhibitor [GC376 positive control from 3CL protease, untagged (SARS-CoV-2) assay kit (BPS Bioscience, San Diego, CA, USA)], and buffer alone (negative inhibition control) were mixed separately with 20 µg of enzymatically active r3CL^pro^. Individual mixtures were added to appropriate wells of a 96-well flat and clear bottom black plate and the plate was kept on an orbital shaker at 25 °C for 30 min. Substrate alone was included as the background control (background). The r3CL^pro^ fluorescent substrate (5 µg) was added to each well and the plate was kept shaking for 4 h. Fluorescent signals (relative fluorescent units, RFU) were recorded at the excitation wavelength 436 nm and emission wavelength 528 nm using Synergy H1 Microplate reader. The 3CL^pro^ activity in the presence of inhibitor/PEN-HuscFvs (test) in relation to the activity of 3CL^pro^ + substrate + buffer (Buffer) was calculated: [(RFU of test-RFU of background)/(RFU of Buffer-RFU of background)].

### 4.14. HuscFv-Mediated Inhibition of the Authentic SARS-CoV-2 Replication

The HuscFv-mediated anti-SARS-CoV-2 assays were performed in BSL-3 facility, Faculty of Medicine Siriraj Hospital, Mahidol University, Bangkok, using the methods described previously [35] with a slight modification. Vero E6 cells were seeded into wells of 24-well-cell culture plates (1.5 × 10^5^ cells/well) and incubated at 37 °C in 5% CO_2_ atmosphere overnight. The cells were added with 500 µL of DMEM with 2% FBS and antibiotics containing 0.25–3.0 µM PEN-HuscFvs or medium alone and kept at 37 °C in 5% CO_2_ atmosphere for 3 h. The fluids in all wells were collected and kept aside. The cells in wells were added with SARS-CoV-2, either Wuhan wild type, B.1.1.7 (α), B.1.351 (β), B.1.617.2 (δ), or B1.1.529 (Omicron) variant (50 pfu in 250 µL per well). After 1 h incubation to allow virus entry, the supernatants were discarded; the kept fluids were added back to the infected cells in the respective wells and incubated for 18 h. The culture fluids and the cells were then collected separately. SARS-CoV-2 RNA in each cell sample was quantified by qRT-PCR while the collected culture fluids containing the SARS-CoV-2 Wuhan wild type and the α, β, and δ variants were subjected to plaque forming assay (PFA) for determining infectious SARS-CoV-2 particles that were released from the infected cells. The omicron variant contained in the culture fluids were determined by focal forming assay (FFA). Two independent experiments were performed.

### 4.15. Quantitative RT-PCR

RNAs were extracted from the cell samples using TRIzol™ reagent (Thermo Fisher Scientific). SARS-CoV-2 RNAs in the cell samples were determined using Brilliant III Ultra-Fast SYBR^®^ Green QRT-PCR Master Mix (Agilent Technologies) and PCR primers (Table S3). The PCR reaction mixture was 10 µL of 2 × SYBR Green qRT-PCR Master Mix, 0.4 µL each of E-forward and E-reverse primers, 0.2 µL of 100 mM DTT, 1 µL of RT/RNase Block, 6 µL of nuclease free water and 2 µL of RNA template. The PCR reaction condition was 50 °C, 10 min; 95 °C, 3 min; and 35 cycles of 95 °C, 5 s and 60 °C, 5 s The fold reduction of the RNAs of the antibody-treated groups compared to the infected cells in medium alone are presented. The EC_50_ values of the PEN-HuscFvs were calculated by using the GraphPad Prism version 9.3 software (GraphPad Software; San Diego, CA, USA).

### 4.16. Plaque Forming Assay (PFA)

Plaque forming assays were performed for determining the number of infectious SARS-CoV-2 particles (Wuhan wild type, α, β, and δ variants) that were released from the antibody-treated infected cells compared to the infected cells in medium alone. Vero E6 cells (1.5 × 10^5^ cells per well) were cultured at 37 °C in 5% CO_2_ incubator overnight in 24 well-culture plates. The cells were rinsed with plain DMEM, added with 10-fold dilutions of the supernatants of the SARS-CoV-2 replication inhibition experiments (tested culture supernatants), and incubated for 1 h. The fluids in all wells were discarded; the infected cells were added with 1.5% carboxymethylcellulose (CMC, Sigma Aldrich, Merck) in DMEM containing 2% FBS and antibiotics and incubated in 5% CO_2_ incubator at 37 °C for 72 h. The cells were fixed with 10% formaldehyde in water at room temperature for 2 h, washed with distilled water (1 mL) five times, and stained with 1% crystal violet in 10% ethanol for 15 min. Excess dye was washed away with distilled water and the plates were air-dried. The number of plaques in all wells was counted visually and the plaque-forming units (pfu) per mL of individual tested culture supernatants were calculated: pfu per mL = plaque number **÷** (infection volume × dilution factor). The results of different treatments were compared.

### 4.17. Focus-Forming Assay (FFA)

Because the SARS-CoV-2 Omicron variant formed tiny plaques, which were difficult to count accurately, the Omicron infectious particles in the supernatants were enumerated using FFA. The supernatants containing the SARS-CoV-2 Omicron variant were diluted 10-fold serially with plain DMEM. Vero E6 cells were seeded into wells of 96 well-culture plates (1 ×10^4^ cells per well) and incubated at 37 °C in 5% CO_2_ incubator overnight. The fluids were discarded and the established Vero E6 in individual wells were added with 50 µL of the diluted supernatants and the plates were incubated at 37 °C in 5% CO_2_ incubator for 1 h. After discarding the fluids from all wells, the infected cells were added with 100 µL of 1.5% CMC in DMEM containing 2% FBS and antibiotics and incubated at 37 °C in 5% CO_2_ incubator for 18 h. Infected cells were fixed with 100 µL of 10% formaldehyde in water at room temperature for 2 h, washed twice with PBS, and permeated with 0.1% Triton-X 100 in PBS at room temperature for 20 min. After washing three times with PBS, the cells were blocked with 3% BSA in PBS, stained with 50 µL of 1:3000 of anti-SARS-CoV-2 N antibody at room temperature for 1 h. After washing with PBS, 1:3000 of HRP-conjugated sondary antibody (50 µL) was added to each well. Fifty microliters of KPL TrueBlue™ Peroxidase Substrate (Cat. No.: 5510-0030, SeraCare) was added and kept in darkness at room temperature for 2-3 h. The foci were observed and counted under inverted microscope (40× magnification) (Nikon Metrology, Brighton, MI, USA). The focus-forming units (FFU) per mL were calculated: FFU per mL = foci number ÷ (infection volume × dilution factor).

### 4.18. Quantification and Statistical Analysis

GraphPad Prism version 9 software (GraphPad Software; San Diego, CA, USA) was used for calculation of the super-antibody EC_50_. GraphPad Prism version 5 (GraphPad Software) was used to compare the results of all tests. Statistically significant difference was determined by one-way ANOVA and Bonferroni test. *P*-values of 0.05 or lower were considered statistically significant. *p* > 0.05 (ns, not significant); *p* ≤ 0.05 (*), *p* ≤ 0.01 (**), *p* ≤ 0.001 (***), *p* ≤ 0.0001 (****).

## Figures and Tables

**Table 1:** SARS-CoV-2 3CL^pro^ domains and residues that formed contact interface with the HuscFv27, HuscFv33 and HuscFv34, and the interactive bonds.

3CL^pro^				HuscFv27		Interactive Bond
Residue	Domain	Function of the Residue		Residue	Domain	
Glu288	III			Arg104	VH-CDR3	Hydrogen and Electrostatic
Asp289	III			Arg104	VH-CDR3	Hydrogen and Electrostatic
Glu 290	III	Homodimerization to form active enzyme		Arg104	VH-CDR3	Hydrogen and Electrostatic
Lys5	N-finger	Interact with Glu166 of another monomer to keep the S1 pocket in correct orientation		Ser103	VH-CDR3	Hydrogen
Lys236	III			Gly42	VH-FR2	Hydrogen
Ala285	III			Asp108	VH-CDR3	Hydrogen
Asn274	III			Gln39	VH-FR2	Hydrogen
Gln273	III			Gly42	VH-FR2	Hydrogen
Met276	III			Tyr95	VH-FR3	Hydrogen
Ser139	II	Conserved residue in proximity of the protease active site		Thr101	VH-CDR3	Hydrogen
Asp289	III			Arg104	VH-CDR3	Hydrogen
Glu290	III	Homodimerization to form active enzyme		Arg104	VH-CDR3	Hydrogen
Leu287	III			Arg104	VH-CDR3	Hydrogen
Glu290	III	Homodimerization to form active enzyme		His105	VH-CDR3	Hydrogen
Ser284	III			Asp108	VH-CDR3	Hydrogen
Gly278	III			Gln235	VL-FR4	Hydrogen
Gly138	II			Gly102	VH-CDR3	Hydrogen
Glu288	III			Phe107	VH-CDR3	Hydrogen
Tyr126	II			Tyr100	VH-CDR3	Hydrophobic
Tyr126	II			Gly102	VH-CDR3	Hydrophobic
Tyr126	II			Ser103	VH-CDR3	Hydrophobic
Leu286	III			Ala97	VH-CDR3	Hydrophobic
Ala285	III			Tyr95	VH-FR3	Hydrophobic
Lys137	II			His105	VH-CDR3	Hydrophobic
**3CL^pro^**				**HuscFv33**		**Interactive bond**
**Residue**	**Domain**	**Function of the Residue**		**Residue**	**Domain**	
Asn51	I			Asp62	VH-FR3	Hydrogen
Lys137	II			Gly102	VH-CDR3	Hydrogen
Glu166	II	Homodimerization to form active enzyme		Tyr59	VH-FR3	Hydrogen
Asp197	II			Ser108	VH-CDR3	Hydrogen
Pro168	II			Thr35	VH-FR2	Hydrogen
Glu166	II	Homodimerization to form active enzyme		Ser52	VH-CDR2	Hydrogen
Gly170	II			Ser52	VH-CDR2	Hydrogen
Glu166	II	Homodimerization to form active enzyme		Ser54	VH-CDR2	Hydrogen
Glu166	II	Homodimerization to form active enzyme		Ser56	VH-CDR2	Hydrogen
Gln189	LLR			Lys65	VH-FR3	Hydrogen
Leu50	I			Lys65	VH-FR3	Hydrogen and Hydrophobic
Asn238	III			Arg107	VH-CDR3	Hydrogen
Asp197	LLR			Ser108	VH-CDR3	Hydrogen
Phe140	II	Homodimerization to form active enzyme		Ser54	VH-CDR2	Hydrogen
Asp197	LLR			Gly102	VH-CDR3	Hydrogen
Thr199	LLR			Ser104	VH-CDR3	Hydrogen
Ala194	LLR			Trp47	VH-FR2	Hydrogen and Hydrophobic
Ala193	LLR			Trp47	VH-FR2	Hydrophobic
Ala191	LLR	Substrate binding		Ala61	VH-FR3	Hydrophobic
Leu286	III			Pro105	VH-CDR3	Hydrophobic
Met165	II	Substrate binding		Tyr59	VH-FR3	Hydrophobic
**3CL^pro^**				**HuscFv34**		**Interactive bond**
**Residue**	**Domain**	**Function of the Residue**		**Residue**	**Domain**	
Glu288	III			Lys177	VL-FR2	Electrostatic
Ser46	I			Gly101	VH-CDR3	Hydrogen
Ser46	I			Asp102	VH-CDR3	Hydrogen
Gly170	II			Asp109	VH-CDR3	Hydrogen
Pro168	II			Trp47	VH-FR2	Hydrogen and Hydrophobic
Asn142	II			Lys98	VH-CDR3	Hydrogen
Thr190	LLR	Substrate binding		Arg99	VH-CDR3	Hydrogen
Ser46	I			Gly104	VH-CDR3	Hydrogen
Glu166	II	Homodimerization to form active enzyme		Phe108	VH-CDR3	Electrostatic
Ala285	III			Ala178	VL-FR2	Hydrophobic
Ala285	III			Pro179	VH-FR2	Hydrophobic
His172	II			Val110	VH-CDR3	Hydrophobic
Ala191	LLR	Substrate binding		Trp47	VH-FR2	Hydrophobic
Ala193	LLR			Trp47	VH-FR2	Hydrophobic
Cys145	II	Catalytic dyad		Trp105	VH-CDR3	Hydrophobic

LLR, long loop region (residues 185–200) that connects domains II and III [22].

**Table 2:** The EC_50_ of the PEN-HuscFv27, PEN-HuscFv33 and PEN-HuscFv34 against SARS-CoV-2.

Clone No.	EC_50_ (nM)				
	Wuhan	α (B.1.1.7)	β (B.1.351)	δ (B.1.617.2)	Omicron (B.1.1.529)
PEN-HuscFv27	692.5	778.1	1058	582.8	2694
PEN-HuscFv33	491.4	696.7	297	478.1	1048
PEN-HuscFv34	539.5	735.9	878.3	158.1	800.9

## Data Availability

The data set or code generated during the present study are accessible from the corresponding author on reasonable request. Request for material can be directed to monrat.chl@mahidol.ac.th or wanpen.cha@mahidol.ac.th. All materials and reagents will be made available upon installment of a material transfer agreement (MTA).

## Supplementary Materials

The supporting information can be downloaded at: https://www.mdpi.com/article/10.3390/ijms23126587/s1.
