# Supplementary material for: Human Superantibodies to 3CLpro Inhibit Replication of SARS-CoV-2 across Variants
Source: Int J Mol Sci. 2022 Jun 13;23(12):6587. doi: 10.3390/ijms23126587 (PMC9223907; doi:10.3390/ijms23126587)
Supplement: Supplementary file 1 [file ijms-23-06587-s001.zip › ijms-1754275-supplementary.pdf]

Supplementary data

Table S1. PCR mixture for 1 reaction

**Component**	**Volume** (μL)
10x *Taq* DNA polymerase buffer with KCl	1.25
25 mM $MgCl_2$	0.75
10 mM dNTPs	0.50
10 μM forward primer	0.25
10 μM reverse primer	0.25
Nuclease free water	9.40
Total	12.5

Table S2. PCR thermal cycles

**Step**	**Temperature** (°C)#	**Time**
Initial denaturation	95	10 min
10 cycles of		
1. Denaturation	95	30 s
2. Annealing	55	30 s
3. Extension	72	1 min per 1 kb
Final extension	72	5 min

Table S3. Oligonucleotide primer sequences used in this study

**Primer**	**Sequence** (5'-3')	**Annealing temperature** (°C)
T7 promotor	TAATACGACTCACTATAGGG	55
T7 terminator	GCTAGTTATTGCTCAGCGG	55
R1	CCATGATTACGCCAAGCTTTGGAGCC	55
R2	GCTAGATTTCAAAACAGCAGAAAGG	55
SARS-CoV-2 E forward	ACAGGTACGTTAATAGTTAATAGCGT	60
SARS-CoV-2 E reverse	ATATTGCAGCAGTACGCACACA	60
